# Seeking or contributing? Evidence of knowledge sharing behaviours in promoting patients’ perceived value of online health communities

**DOI:** 10.1111/hex.13146

**Published:** 2020-10-13

**Authors:** Cui Guo, Zhen Zhang, Junjie Zhou, Zhaohua Deng

**Affiliations:** ^1^ Shantou University Business School Shantou China; ^2^ Huazhong University of Science & Technology Wuhan China

**Keywords:** health knowledge sharing, health services, online health communities, social interaction, social support, telemedicine

## Abstract

**Background:**

Health knowledge, as an important resource of online health communities (OHCs), attracts users to engage in OHCs and improve the traffics within OHCs, thereby promoting the development of OHCs. Seeking and contributing health knowledge are basic activities in OHCs and are helpful for users to solve their health‐related problems, improve their health conditions and thus influence their evaluation of OHCs (ie perceived value of OHCs). However, how do patients’ health knowledge seeking and health knowledge contributing behaviours together with other factors influence their perceived value of OHCs? We still have little knowledge.

**Objective:**

In order to address the above gap, we root the current study in social cognitive theory and prior related literature on health knowledge sharing in OHCs and patients’ perceived value. We treat health knowledge seeking and health knowledge contributing behaviours as behavioural factors and structural social capital as an environmental factor and explore their impacts on patients’ perceived value of OHCs.

**Design:**

We have built a theoretical model composed of five hypotheses. We have designed a questionnaire composed of four key constructs and then collected data via an online survey.

**Setting and participants:**

We have distributed the questionnaire in two Chinese OHCs. We obtained a sample of 352 valid responses that were completed by patients having a variety of conditions.

**Results:**

The empirical results indicate that health knowledge seeking and health knowledge contributing have positive impacts on patients’ perceived value of OHCs. The impact of health knowledge seeking on patients’ perceived value of OHCs is greater than the impact of health knowledge contributing. In addition, structural social capital moderates the effects of health knowledge seeking and health knowledge contributing on patients’ perceived value of OHCs. It weakens the effect of health knowledge seeking but enhances the effect of health knowledge contributing on patients’ perceived value of OHCs.

**Conclusions:**

These findings contribute to the literature on patients’ perceived value of OHCs and on the role of structural social capital in OHCs. For OHC managers, they should provide their users more opportunities to seek or contribute health knowledge in their communities.

## 1 INTRODUCTION

As information and communication technologies (ICTs) have become ubiquitous, people have become increasingly active in online health‐related applications such as online health communities (OHCs). OHCs are a type of health‐related virtual community (VC) designed particularly for different health‐related stakeholders, for example health insurance, pharmaceutical companies, hospitals, health professionals, patients, and patients’ relatives or friends.^1^, ^2^, ^3^, ^4^, ^5^, ^6^, ^7^, ^8^, ^9^ There are different types of OHCs in which users can conduct different health‐related activities, including transactions, appointment scheduling, counselling, social networking and health‐related Q&As.^1^, ^10^, ^11^, ^12^, ^13^, ^14^, ^15^, ^16^ In this study, we particularly focus on problem‐solving communities where both health professionals and patients can participate and collaborate for health knowledge exchange, for example Q&A forums on health conditions,^13^, ^17^ mental health‐focused Q&A forums,^18^, ^19^ pregnancy forums such as Babytree.com,^20^ and cancer‐focused communities.^3^, ^21^, ^22^ In this type of OHCs, health professionals can provide professional health knowledge by contributing to the community and responding to patients’ health‐related questions.^20^, ^23^ Patients can disclose their personal health conditions, make new social ties, and seek or contribute health knowledge.^1^, ^6^, ^24^, ^25^ Using OHCs can help health professionals build their reputations and earn material rewards^12^, ^13^, ^19^, ^26^ and help patients improve their health outcomes, such as their e‐health literacy and feeling of well‐being.^27^, ^28^, ^29^ These advantages make OHCs an effective way to alleviate the pressures on medical resources.^30^, ^31^

Exchanging health knowledge and information is a kind of basic activity in OHCs.^3^, ^8^, ^9^, ^20^, ^21^, ^32^, ^33^, ^34^ Health knowledge in OHCs is a public good, and contributors lose their control over the knowledge they shared.^35^, ^36^, ^37^ Scholars thus are curious about the reasons why people contribute health knowledge in OHCs. For example, some studies on health professionals have examined the impacts of factors such as professional capability, reputation and economic rewards.^7^, ^8^, ^19^, ^20^, ^31^, ^38^, ^39^ Other studies, focused on patient users, have examined the impacts of extrinsic and intrinsic motivations^4^, ^5^, ^6^, ^20^, ^33^, ^34^, ^40^ and potential hindering factors such as trust and privacy protection.^6^, ^24^, ^30^ As social networking is an important feature of OHCs and users in OHCs also pursue social interactions,^1^, ^3^, ^22^ some studies have also explored the impacts of users’ social capital in OHCs.^13^, ^28^, ^29^

In addition to exploring its antecedent factors, scholars recently began to explore the health outcomes of exchanging health knowledge in OHCs. For example, knowledge seekers can obtain health knowledge for their health issues and then use it to improve their health conditions.^9^, ^41^, ^42^ Knowledge contributors also can obtain new knowledge because they have to understand other questions and then combine different knowledge to address those questions^9^, ^29^; this process could help them to create new knowledge. OHC use therefore positively promotes users’ health outcomes such as health conditions, health attitude and e‐health literacy.^28^, ^29^, ^43^ As a kind of health outcome, users’ perceived value is a crucial antecedent for users’ satisfaction with OHCs and also their continuous use of OHCs.^44^, ^45^ In this paper, we will clarify how users’ perceived value of OHCs is a crucial antecedent of their satisfaction with OHCs, their continuous use of OHCs and related health outcomes in section Literature Review. As discussed above and in section Literature Review, few studies have explored how health knowledge exchanging behaviours and other factors influence users’ perceived value of OHCs.

In order to address the above gap, we adopted social cognitive theory (SCT) as theoretical foundation. We treated health knowledge seeking and health knowledge contributing as behavioural factors and structural social capital as a social environmental factor, and, finally, built a model composed of five hypotheses. We tested our hypotheses with a sample of 352 valid responses.

## 2 LITERATURE REVIEW

### 2.1 Social cognitive theory

Social cognitive theory (SCT) is a classical theory on individual behaviours. According to SCT, personal behaviours are shaped by the factors from three domains (ie environment, cognition and behaviour); the factors from any two domains can interact with each other and then influence the factors in the third domain.^46^ For example, interactions between environmental and behavioural factors, which can be treated as parts of social environments, influences an individual's cognitions and, in turn, reshapes their behaviours and external environment.^46^

In addition to being used to explain personal knowledge sharing behaviours in VCs,^37^, ^40^, ^47^, ^48^, ^49^ SCT also has been used to analyse change in personal cognition. For example, environmental factors such as trust and interaction positively influence personal cognitive factors such as outcome expectation.^37^, ^48^ Personal health knowledge seeking behaviours together with environmental factors (eg structural social capital) positively influences cancer survivors’ e‐health literacy.^28^ The above studies indicate that the change in personal cognitive factors could be explained by SCT. Since we focus on how behavioural factors and environmental factors influence patients’ perceived value of OHCs, we therefore adopt SCT as a theoretical foundation. We propose that patients’ knowledge sharing behaviours (ie behavioural factors) together with their structural social capital (ie social environmental factor) influence patients’ perceived value of OHCs.

### 2.2 Health knowledge sharing in OHCs

Following prior studies,^37^, ^49^, ^50^, ^51^ we define health knowledge sharing as a process composed of two aspects: health knowledge seeking and health knowledge contributing. Health knowledge in OHCs includes people's physical health, mental health, diseases and nutrition, such as hospital or doctor information, healthy life and behaviours, medicine information, personal health conditions, medical treatments and medical experiences.^34^, ^52^ Health knowledge seeking refers to the search, acquisition or consumption of health knowledge in OHCs.^51^ Health knowledge contributing refers to the generation or provision of health knowledge in OHCs.^53^ We reviewed prior studies on health knowledge sharing in OHCs and summarize the results in Table 1.

**Table 1:** The sampling of research on knowledge sharing behaviours within OHCs

References	Objects	Dependent variable(s)	Independent variable(s)
^9^	Patient users	Health conditions	Information support given (+), information support received (+), emotional support given (+), emotional support received (+)
^34^	Patient users	General health knowledge contributing	Sense of self‐worth (+), reputation (+), social support (+), face concern (+), executional costs (‐)
		Specific health knowledge contributing	Sense of self‐worth (+), reputation (+), social support (+), face concern (‐), cognitive costs (‐)
^20^	Patient users	Health knowledge contributing	Knowledge self‐efficacy (ns), altruism (+), empathy (+), reputation (ns), reciprocity (+)
	Health professionals		Knowledge self‐efficacy (+), altruism (+), empathy (ns), reputation (+), reciprocity (+)
^33^	Patient users	Health knowledge contributing	Perceived benefits (+), perceived risks (ns)
		Health knowledge seeking	Perceived benefits (+), perceived risks (ns)
^29^	Patient users	Health literacy	Information support provisioning (+), Information support receipt (+)
		Health attitude	Emotional support provisioning (+), Emotional support receipt (+)
^19^	Health professionals	Voluntary participation behaviours	Technical competence (TC, +), online reputation (OR, +), economic rewards (ER, +), TC*OR (‐), TC*ER (‐)

Note

Relationships between independent variables and dependent variables are shown after each independent variable (ns: not significant; +: positive; ‐: negative).

As shown in Table 1, early research primarily focused on the antecedent factors influencing users’ health knowledge sharing behaviours. Since meeting users’ expectations and enriching their health outcomes are critical for users’ continuous use of OHCs and the sustainability of OHCs,^7^, ^19^, ^20^, ^54^ recent studies have begun to explore the consequences of their health knowledge sharing behaviours, for example the impacts of informational support in OHCs on patients’ health conditions, health attitude and e‐health literacy.^9^, ^28^, ^29^, ^43^, ^55^ Although engaging in OHCs can improve users’ perception of value,^29^, ^56^ few studies have examined the impacts of patients’ health knowledge sharing behaviours and other factors on their perceived value of OHCs. This study aimed to address the above gap.

### 2.3 Patient social capital

Social capital is defined as the sum of the actual and potential resources that an individual obtains from the network of relationships.^57^ Social capital can be divided into three dimensions: structural social capital, relational social capital and cognitive social capital.^57^ OHCs are online health‐related social networks in which users with common interests, goals or practices interact to contribute and seek health knowledge and engage in social interactions.^1^, ^26^, ^47^ It is the nature of social interactions and the resources embedded in social interaction networks that sustain the OHCs.^26^, ^47^ Therefore, in addition to health knowledge resources, users’ structural social capital developed from social interactions. This is crucial and most relevant within OHCs.^4^, ^29^, ^47^, ^58^ We thus incorporate structural social capital and explore its impacts on patients’ perceived value of OHCs. Specifically, structural social capital in this study refers to the overall pattern of connections such as the strength of relationships, the level of time spent and/or the frequency of the interactions among individuals in OHCs.^47^, ^59^ We summarize prior related studies on social capital in OHCs in Table 2.

**Table 2:** Prior studies on social capital in OHCs

References	Dependent variable(s)	Independent variable(s)
^60^	Social support	Structural social capital (+)
^4^	Knowledge externalization	Structural social capital (+), relational social capital (ns), cognitive social capital (ns)
	Knowledge combination	Structural social capital (+), relational social capital (ns), cognitive social capital (ns)
^23^	Knowledge contributing	Social capital (+)
^28^	E‐health literacy	Structural social capital (+)
^29^	Informational support exchange	Structural social capital (+)
	Emotional support exchange	Structural social capital (+)
^21^	Informational support	Structural social capital (ns), relational social capital (ns), cognitive social capital (+)
	Emotional support	Structural social capital (+), relational social capital (+), cognitive social capital (+)
	Companionship support	Structural social capital (+), relational social capital (+)

Note

Relationships between independent variables and dependent variables are shown after each independent variable (ns: not significant; +: positive; ‐: negative).

Prior studies have explored the impacts of social capital on users’ health expectations by OHCs use. For example, structural social capital can alleviate patients’ perceived stress, depression and coping,^60^ and enhance patients’ e‐health literacy.^28^ Structural social capital also can improve patients’ health literacy and attitude via facilitating patients’ social support provisioning and receipt in OHCs.^29^ Some scholars also have examined the direct and mediating effects of structural social capital on user‐perceived value.^56^, ^61^, ^62^ For example, Lee et al (2014) have found that structural social capital indirectly creates perceived value through information contributing behaviours.^56^ Zhang et al (2017) have verified that structural social capital can positively influence perceived value and then indirectly influence users’ continuance intention to use WeChat.^61^ Similarly, Luo and Ye (2019) have examined the direct effect of structural social capital on user‐perceived value in VCs.^62^

As discussed above, prior studies primarily focus on the direct effects on users’ health expectations^19^, ^29^, ^60^ and recently have begun to explore its moderating effects.^28^ This study follows the above trend. We treat structural social capital as an environmental factor and will examine its direct and moderating effects on patient perceived value of OHCs.

### 2.4 Patients’ perceived value

Perceived value of OHCs is defined as patients’ perception of the overall utility based on a trade‐off between perceived benefits and costs of using OHCs.^63^ Studies considering the direct studies on patients’ perceived value of OHCs are few, so we summarized prior related studies on user‐perceived value for our reference in this study (see Table 3).

**Table 3:** Prior studies on user‐perceived value in IS

References	Contexts	Perspective(s)	Dependent variable(s)	Independent variable(s)
^65^	Knowledge management systems	DeLone and McLean's IS success model	Perceived benefits	System quality (+), knowledge quality (+), system use (ns)
^68^	Virtual P3 community	Social capital	Information value, social value	Social capital (+)
^67^	Mobile services	DeLone and McLean's IS success model	Perceived value	Service quality (+)
^64^	Transactional virtual communities	Cost–benefit	Perceived net goal attainment	Extrinsic benefit (+), intrinsic benefit (+), opportunity cost (‐), Actual cost (ns)
^66^	Mobile services	Technology acceptance model	Perceived value	Perceived ease of use (ns), perceived usefulness (+), mobility (+), perceived security (+)
^44^	Virtual communities	Resource‐based view	Perceived value	Relationship resources (+), technology infrastructure (+), knowledge resources (+), human resources (+)
^56^	Social medias	Social capital	Information value, experiential value, transaction value, social value	Information contributing (+)

Note

Relationships between independent variables and dependent variables are shown after each independent variable (ns: not significant; +: positive; ‐: negative).

As shown in Table 3, the factors influencing user‐perceived value can be categorized into two domains, that is individual factors and environmental factors. Individual factors include personal factors such as benefits and costs,^64^ information contributing^56^ and system use behaviours.^65^ Environmental factors are mainly related to the external social environment, such as perceived usefulness,^66^ quality factors,^65^, ^67^ resource factors^44^ and social capital.^68^

Users’ behaviours positively influence their perceived value.^56^, ^65^, ^69^, ^70^, ^71^, ^72^, ^73^ For example, users’ engagement in VCs can improve their perception of value including expanded social relationships^69^, ^73^ and functional needs.^73^ In VCs, community users can develop the perception of value through health/general topic interactions with other users.^71^, ^72^, ^73^ Besides, knowledge contributing behaviours can not only help other users to solve problems, but also are beneficial for contributors to perceive value including improved emotions and new close relationships.^56^, ^70^, ^72^ Although social capital as environmental factors positively influence users’ health outcomes,^13^, ^28^, ^29^ few studies have explored how structural social capital influences patients’ perceived value of OHCs.

## 3 RESEARCH MODEL AND HYPOTHESIS DEVELOPMENT

**Figure 1:**
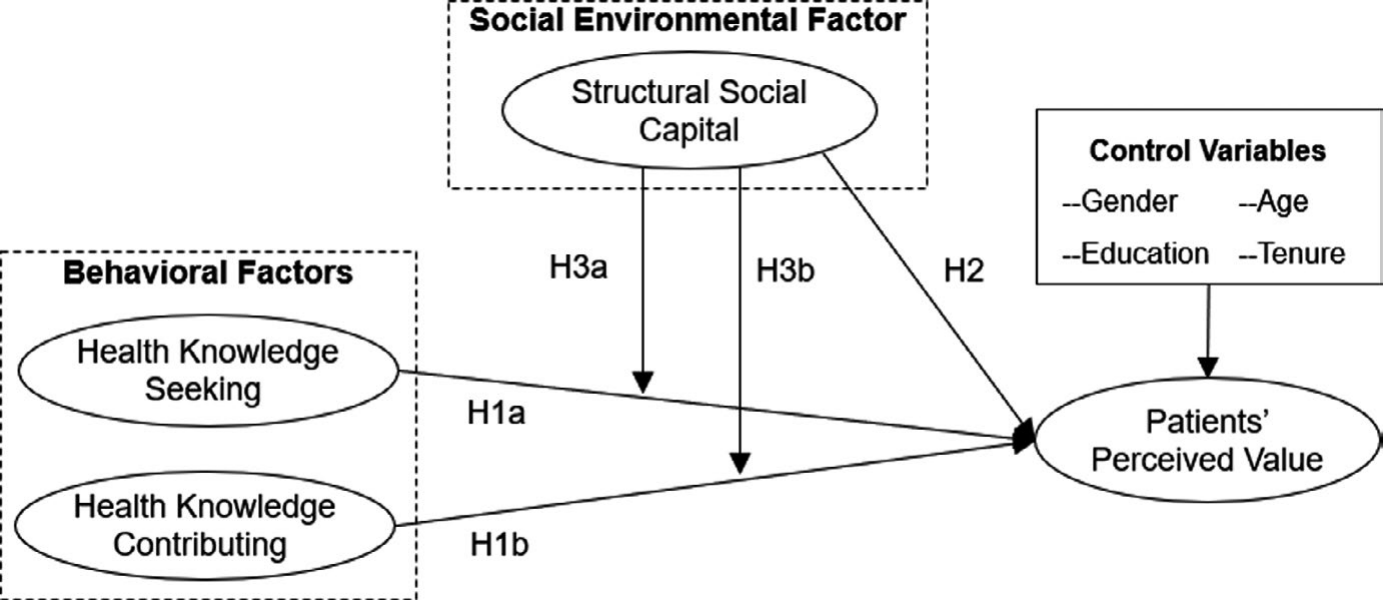
Research model and hypotheses

This study aimed to examine how patients’ knowledge sharing behaviours and their structural social capital influenced their perceived value of OHCs. Specifically, we conceptualized health knowledge seeking and health knowledge contributing as behavioural factors and structural social capital as a social environmental factor. We proposed that both environmental factors and behavioural factors directly influence patients’ perceived value; in addition, structural social capital as a social environmental factor moderates the effects of behavioural factors on patients’ perceived value of OHCs. The proposed research model is shown in Figure 1.

### 3.1 Main effects

Health knowledge seeking is defined as users’ search, acquisition or consumption of health knowledge in OHCs.^51^ Under the user‐generated content mechanism, health professionals and patient users can collaborate with each other and contribute health knowledge into OHCs.^20^ OHC users, especially patients with health issues, can search for health information or post directly for help within OHCs. They can seek and obtain the health knowledge they need.^9^ They can then use professional health knowledge to better understand their health conditions, seek possible treatment solutions, conduct self‐management activities^41^ and reduce disease risks.^42^ Therefore, health knowledge seeking behaviour in OHCs is beneficial for patients to improve their health outcomes and make them feel that using OHCs is worthwhile. We thus hypothesized that,

H1a: Health knowledge seeking behaviour has a positive impact on patients’ perceived value of OHCs

Health knowledge contributing refers to patients’ generation or provision of health knowledge in OHCs.^53^ Health knowledge contributing behaviour gives the contributors a feeling of value in two ways. First, knowledge contributors need to understand help seekers’ questions and then contextualize their knowledge to generate better answers before posting health knowledge into OHCs. Such a process enhances the contributors’ understanding of health knowledge^29^ and supports their learning of new knowledge in this collaborative consumption process.^9^ Second, health knowledge contributing behaviours enriches health knowledge in OHCs and meets seekers’ needs of health knowledge that is useful to solve their health‐related issues.^41^ Health knowledge seekers in turn are more likely to express their gratitude to the contributors.^29^ In above process, contributors could develop close relationships with other users and obtain a sense of self‐worth from other users’ gratitude.^20^, ^23^, ^34^ Namely, health knowledge contributing behaviour is beneficial for users to improve their evaluation of the utility of OHC use (ie perceived value). We thus hypothesized that,

H1b: Health knowledge contributing behaviour has a positive impact on patients’ perceived value of OHCs

Structural social capital describes the strength of relationships, the level of time spent and/or the frequency of the interactions among patients in the same OHC.^47^, ^59^ Structural social capital acts as a social environmental factor and provides patients opportunities to evaluate the potential value they could provide. For those patients who have a higher level of structural social capital, they can utilize these opportunities to maximize their expected outcomes.^61^, ^74^, ^75^ The more frequently the patients engage in OHCs, the more likely they better understand the health knowledge that is useful for their health issues,^76^, ^77^ improve their health conditions^29^, ^60^ and expand social relationships through interacting with others.^61^ Considering that patients’ perceived value refers to the total utility derived from their solved health issues, improved health conditions and expanded social relationships, structural social capital thus has a positive effect on patients’ evaluation of OHC experience (ie perceived value of OHCs).^78^ Hence, we hypothesized that,

H2: Structural social capital has a positive impact on patients’ perceived value of OHCs

### 3.2 Moderating effects

According to SCT, individuals’ behaviours together with environmental factors can reshape their cognitions.^46^ OHCs enable knowledge seekers to obtain relevant knowledge to improve their health conditions.^42^ High structural social capital usually means users have more social contacts. When seeking health knowledge in OHCs, patients with high structural social capital may receive massive replies and the useful knowledge, thus might be overwhelmed by those that are useless.^79^ They need to devote a lot of time and energy to distinguish useful information from useless ones and are more likely to experience negative emotions such as anxiety and depression.^80^ The added unnecessary costs would make them underestimate their perceived value of OHC use. In addition, higher structural social capital also means that patients have diversified channels to seek their needed health resources and obtain more value.^57^ They can find what they need through other activities such as through personal channels instead of through public postings. When users use more personal channels to seek knowledge, they will rely less on health knowledge seeking in OHCs. Their perceived value of OHC use derived from health knowledge seeking thus is weakened by structural social capital. We hypothesized that,

*H3a: Structural social capital weakens the impact of patients’ knowledge seeking behaviours on their perceived value of OHCs: when structural social capital is high, the effect of knowledge seeking behaviours will be weaker, else will be stronger*.

As a social environmental factor, structural social capital provides patients new channels to interact with each other.^47^, ^59^ They therefore have more opportunities to discuss health knowledge and collaborate with others to generate new health knowledge. For health knowledge contributors with a higher level of structural social capital, their knowledge could be exposed to more users and therefore receive more gratitude. The positive feedback and experience obtained in above process will enhance contributors’ sense of self‐worth.^20^, ^23^, ^34^ Their perceived value of OHC use derived from health knowledge contributing behaviours thus will be enhanced. We thus hypothesized that,

*H3b: Structural social capital enhances the impact of health knowledge contributing on patients’ perceived value of OHCs: when structural social capital is high, the effect of knowledge contributing behaviours will be stronger*.

In addition to the above variables, prior studies have found that women are more likely to continue participating in sharing,^81^ age has a negative effect on users’ participation behaviours in VCs,^21^ education has positive effect on users’ health knowledge contribution,^23^ and tenure has positive effect on users’ information‐seeking behaviours in VCs.^82^ We thus proposed that gender, age, education and tenure also might influence patients’ perceived value of OHCs, and we treated them as control variables.

## 4 METHODOLOGY

We designed a questionnaire and an online survey for data collection and hypothesis test. This research was approved by the Shantou University Academic and Ethics Board.

### 4.1 Constructs and scales

All scales for our four key constructs were adopted from prior research and adapted to the OHC context. We took the following precautions to translate the English scales into Chinese with an iteration to ensure the meanings of the scale in English and in Chinese were consistent. First, the second author together with two graduate students translated all construct scales into Chinese and did necessary iterations to make a draft Chinese version. Second, the first author and third author who are bilinguals further checked the draft version and made necessary changes to make sure the meaning of all constructs in English and in Chinese converged. Third, in order to make sure all measurement items were clear and understandable, we also did a pilot study by inviting 12 undergraduate students who have OHC use experience to complete the questionnaire. During the process, we asked them to tell us any confusing issues and then modified them accordingly. The questionnaire was frozen when the back‐and‐forth translation and pilot test were completed. We used a 5‐point Likert‐type scale (note: 1 for completely disagree and 5 for completely agree). Table 4 shows the final items of all constructs.

**Table 4:** Scales for constructs

Constructs	Items	Sources
Health knowledge seeking (HKS)	I often use the online health community to seek health knowledge	^51^
	I frequently use the online health community to seek health knowledge	
	I spend a lot of time using the online health community to seek health knowledge	
Health knowledge contributing (HKC)	I frequently participate in health knowledge sharing activities in the online health community	^53^
	I usually spend a lot of time conducting health knowledge sharing activities in the online health community	
	When participating in the online health community, I usually actively share my health knowledge with others	
	When discussing a complicated issue, I am usually involved in the subsequent interactions	
	I usually involve myself in discussions of various topics rather than specific topics	
Patient structural social capital (PSC)	I maintain close social relationships with some members in the online health community	^59^
	I spend a lot of time interacting with some members in the online health community	
	I know some members in the online health community on a personal level	
	I have frequent communication with some members in the online health community	
Patients’ perceived value (PPV)	I think it is a good value for the money to use the online health community	^63^
	I think the cost of using the online health community, such as money, time, and effort, is acceptable	
	I think the product/service of the online health community is considered to be a good buy	

### 4.2 Data collection

Data were collected via an online survey in two Chinese OHCs, that is Mijian (note: Mijian means *Seeking Health*, www.mijian360.com) and Yuaigongwu (note: Yuaigongwu means *Dancing with Cancers*, www.yuaigongwu.com). We clearly informed all participants that the survey was voluntary, and all data would be used only for academic research. We added two questions ('Have you ever used OHCs?' and 'Please write down the name of the OHC you use most frequently') to determine whether the participant had ever used OHCs. If they had never used an OHC, the survey ended. Each participant could respond to the questionnaire only once. The survey began on 28 January 2019 and lasted for 41 days. After deleting 24 invalid items (eg all questions were answered with the same answer, or the respondent failed to identify the reverse question), we obtained a sample of 352 valid responses. Based on the sample of 352 valid responses, we conducted descriptive statistics, assessed the measurement model and tested the structural model.

## 5 RESULTS

### 5.1 Results of descriptive statistics

Table 5 shows the results of demographic statistics.

**Table 5:** Demographic statistics of the samples

		Freq.	Per. (%)			Freq.	Per. (%)
Gender	Male	97	27.6	Education	High school and below	116	33.0
	Female	255	72.4		College	79	22.4
					Undergraduate	122	34.7
Age	<16	1	0.3		Post‐graduate and above	35	9.9
	16‐25	59	16.8	Tenure*	<1	158	44.9
	26‐35	89	25.3		1‐2	102	29.0
	36‐45	86	24.4		2‐3	47	13.4
	46‐55	80	22.7		3‐4	22	6.3
	>55	37	10.5		4‐5	5	1.4
					>5	18	5.1

Note

* Tenure refers to the user's registered history in an OHC and measured by the time a user has been a member of an OHC.

There were significantly more female respondents (255 out of 352) than male ones. We checked this over‐representation of females with the website Mijian. This ratio is appropriate, because many respondents use OHCs due to gender‐related illnesses, such as breast cancer. Over 89% of participants ranged in age from 16 to 55 years. Participants aged from 26 to 35 years account for the highest proportion (25.3%). In addition, over 55% of participants have OHC use experience of more than one year. Of all users in this study, about 67% have a college‐level or higher education. This suggests that users with higher education levels have a higher tendency to use OHCs for health knowledge.^83^

### 5.2 Results of measurement model assessment

We assessed the measurement model with explorative factor analysis using SPSS 20 (see Table 6) and confirmative factor analysis using Mplus 7.4 (see Table 7).

**Table 6:** Factor loadings

Items	HKS	HKC	PSC	PPV
HKS1	**0.575**	0.108	0.120	0.547
HKS2	**0.810**	0.163	0.165	0.383
HKS3	**0.879**	0.199	0.214	0.109
HKC1	0.224	**0.764**	0.365	0.142
HKC2	0.295	**0.743**	0.386	0.091
HKC3	0.047	**0.802**	0.321	0.306
HKC4	0.153	**0.800**	0.325	0.206
HKC5	0.072	**0.819**	0.284	0.175
PSC1	0.206	0.356	**0.769**	0.225
PSC2	0.261	0.448	**0.737**	0.118
PSC3	0.131	0.390	**0.799**	0.189
PSC4	0.118	0.427	**0.823**	0.182
PPV1	0.164	0.229	0.103	**0.831**
PPV2	0.218	0.113	0.215	**0.737**
PPV3	0.124	0.195	0.136	**0.830**
Cronbach's α	0.843	0.933	0.857	0.931

Note

HKS, HKC, PSC and PPV are abbreviations for health knowledge seeking, health knowledge contributing, patient structural capital and patients’ perceived value, respectively.

**Table 7:** Correlation matrix

	Mean	SE	SD	CR	AVE	AVE square root	HKS	HKC	PSC	PPV
HKS	3.558	0.051	0.956	0.859	0.673	0.821	1			
HKC	3.06	0.054	1.008	0.928	0.720	0.848	0.467***	1		
PSC	2.873	0.057	1.067	0.932	0.774	0.880	0.492***	0.787***	1	
PPV	4.072	0.04	0.752	0.849	0.653	0.808	0.557***	0.470***	0.451***	1

Note

Values in bold refer to the square root of AVE; *P < .05, **P < .01, ***P < .001.

For convergent validity, as shown in Table 6, all the factor loading values are greater than 0.5 and all average variance extracted (AVE) values are greater than 0.5, indicating most variances are successfully extracted. In addition, the composite reliability (CR) values are all greater than 0.7 and all Cronbach's α values are greater than 0.6. These indices indicate the convergent validity is good.

For discriminant validity, as shown in Table 6, all the item loadings in their respective factors are greater than the value in their irrespective factors. For example, the factor loadings of four PSC items on PSC (ie respective construct) are above 0.737 and on HKS, HKC and PPV (ie irrespective constructs) are less than 0.448; the former values are greater than the later ones, indicating good discriminant validity. In addition, as shown in Table 7, the AVE square root value of one variable is greater than the correlation value between this variable and the other three variables. These indices indicate a good discriminant validity.

We also checked the potential collinearity issues in three different ways. First, the eigenvalue of every single independent variable is not equal to 0 and the greatest conditional index value is 3.289 that is less than 20,^84^ Second, the greatest variance inflation factor (VIF) value is 2.784 which is less than the suggested value 10.^85^ Third, the correlation value between health knowledge contributing and structural social capital is 0.787 which is less than the cut‐off value 0.8.^86^ Therefore, the multicollinearity has no serious effect on the empirical results.

We also tested the model fitness (see Table 8). All indices are at or over the acceptable level, indicating the model fitness is good.^37^

**Table 8:** Mode‐fit indexes for measurement model

Indexes	Χ^2^	*df*	Χ^2^/*df*	GFI	AGFI	NFI	CFI	SRMR	RMSEA
Results	220.729	80	2.759	0.917	0.875	0.951	0.968	0.042	0.071
Criteria	—	—	= < 5 ^92^	> = 0.9 ^93^	> = 0.8 ^93^	> = 0.9 ^92^	> = 0.9 ^92^	= < 0.08 ^94^	= < 0.08 ^94^

### 5.3 Results of structural model assessment

**Figure 2:**
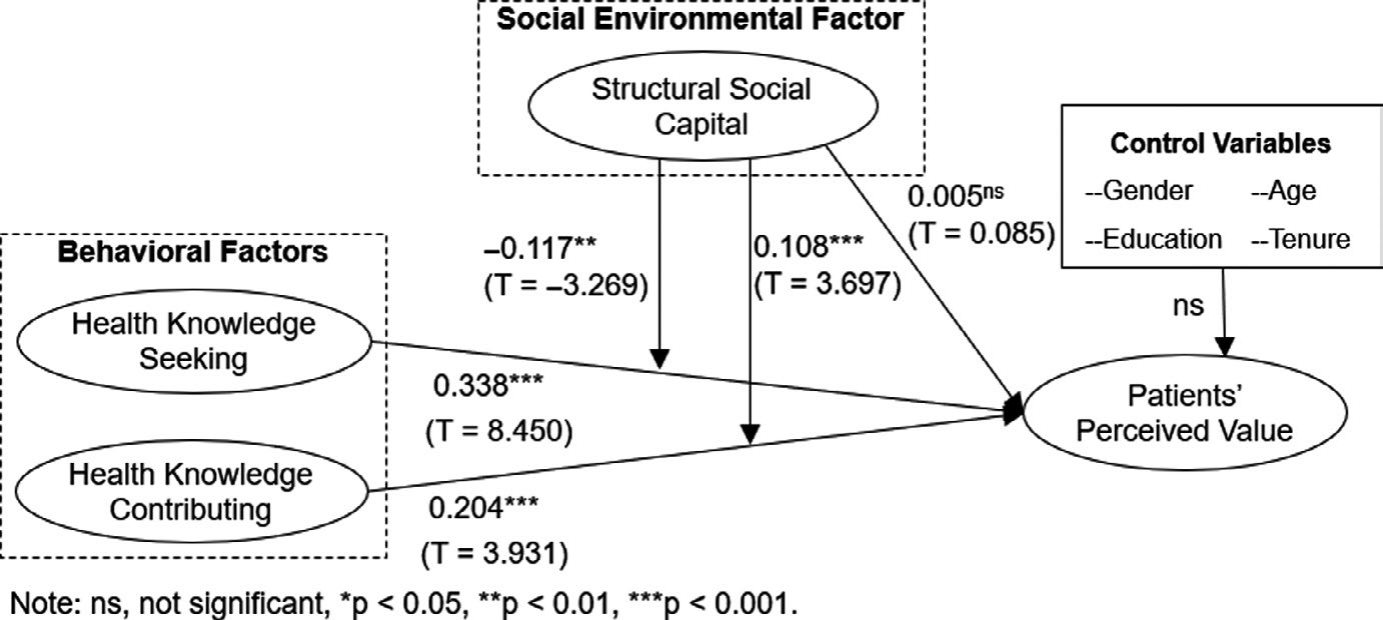
Results for the research model

Although the correlation value among different variables meets the cut‐off value 0.8,^86^ they are still slightly high. In such a situation, structural equation modelling using latent variables works better.^87^, ^88^ We therefore used the latent moderated structural equations (LMS) approach via Mplus 7.4 to test all hypotheses (see Figure 2).

As shown in Figure 2, the effects of health knowledge seeking (β = 0.338, T value = 8.450) and health knowledge contributing (β = 0.204, T value = 3.931) on patients’ perceived value are significant. H1a and H1b are supported. Namely, seeking or contributing health knowledge behaviours can improve patients’ perceived value of OHCs. We further compared the coefficient difference between health knowledge seeking and health knowledge contributing on perceived value of OHCs with a bootstrapping procedure in Mplus 7.4.^89^ The test results show the difference between coefficient estimates of health knowledge seeking and health knowledge contributing is significant (*P* = .043, T = 2.027). Namely, the coefficient of health knowledge seeking is significantly greater than the coefficient of health knowledge contributing; seeking knowledge directly meets patients’ health needs and brings patients a higher sense of value.

The moderating effects of structural social capital on health knowledge seeking (β = −0.117, T value = −3.269) and health knowledge contributing (β = 0.108, T value = 3.697) are significant. H3a and H3b are supported. Namely, patients’ structural social capital weakens the effect of health knowledge seeking but strengthens the effect of health knowledge contributing on patients’ perceived value.

The direct effect of structural social capital on patients’ perceived value is not significant (β = 0.005, T value = 0.085). H2 is unsupported. Therefore, the social environment does not directly improve patients’ perceived value of OHCs. However, structural social capital acts as a moderator that changes the effects of patients’ knowledge sharing and knowledge contributing behaviours.

Finally, no control variables had a significant effect. No gender, age, education and tenure differences were shown. Patients’ perceived value is therefore related to factors other than personal demographic variables.

## 6 DISCUSSION

We have examined how health knowledge seeking and health knowledge contributing behaviours and structural social capital influence patient perceived value of OHCs with a sample of 352 valid responses. Four out of the five hypotheses were supported. As the empirical results show, health knowledge seeking and health knowledge contributing have positive impacts on patients’ perceived value of OHCs; the impact of health knowledge seeking on patients’ perceived value of OHCs is greater than the impact of health knowledge contributing. In addition, structural social capital weakens the effect of health knowledge seeking but enhances the effect of health knowledge contributing on patients’ perceived value of OHCs. In contrast to our predictions, hypothesis H2 was unsupported. This finding is controversial with a prior conclusion that individuals who have more structural social capital are more possibly perceiving a high level value.^61^, ^68^, ^75^ We found some evidence to explain this different finding. For example, one study found that structural social capital positively influences information quantity but does not significantly influence information quality.^79^ Namely, structural social capital does not necessarily indicate high‐quality knowledge. Thus, it is reasonable to find that structural social capital cannot significantly impact patients’ perceived value of OHCs, because patients’ perceived value is more likely linked to health knowledge quality rather than a high quantity of possibly misleading information.^90^ This finding shows that the impacts of structural social capital on user‐perceived value might be more complex than was previously thought. More studies are needed to examine this finding.

### 6.1 Contributions to research

Our findings make two significant theoretical contributions. First, we contribute to the literature on user‐perceived value of OHCs by verifying the impacts of patients’ health knowledge sharing behaviours. Our empirical results indicate that health knowledge seeking and knowledge contributing behaviours positively influence patients’ perceived value of OHCs. This finding is similar to prior conclusions that knowledge seeking and knowledge contributing behaviours enabled individuals to obtain a higher level of perceived value^56^ and that using OHCs helped patients obtain necessary information to improve their health conditions.^9^, ^28^, ^29^, ^43^ In addition, the coefficient of health knowledge seeking is greater than the coefficient of health knowledge contributing, indicating that health knowledge seeking activities contribute more to patients’ perceived value than knowledge contributing activities do. This finding differs from Chen et al’s (2019) finding that health knowledge contributing had a stronger impact on patient health conditions than health knowledge seeking did. This difference might be relevant to patients’ primary purpose of participating in OHCs. When their purpose is to ask for help and obtain health information to solve their health‐related problems, patients can directly meet their needs via knowledge seeking behaviours with less costs and thus have a higher feeling of perceived value.^91^

Second, we contribute to the literature on the roles of environmental factors by having verified a new moderator (ie structural social capital). Our empirical results indicate that patients’ structural social capital enhances the impact of health knowledge contributing but weakens the impact of health knowledge seeking on patients’ perceived value of OHCs. We provide two explanations on the negative moderating role of structural social capital. First, patients with high structure social capital have diversified channels to obtain needed health knowledge in OHCs.^21^, ^57^ They can meet their needs through health knowledge seeking or other personal channels, such as directly asking friends for help. In addition, because interactions via texts in OHCs are asynchronous, patients must wait for answers. Health knowledge seeking activities are thus time‐consuming. Patient structural social capital thus weakens the relationship between health knowledge seeking and patients’ perceived value of OHCs. Second, when seeking health knowledge in OHCs, patients with high structural capital may receive massive replies. They may then face the problem of having too much information,^79^ requiring them to put more effort into distinguishing useful replies from useless ones. In such a situation, patients with high structural social capital are more likely to experience negative emotions.^80^ Structural social capital thus has a negative moderating effect on the relationship between health knowledge seeking and patients’ perceived value of OHCs.

### 6.2 Implications for practice

This study makes several contributions to OHC practice. First, OHC could be used as channels for medical education. As our empirical results show, patients’ perceived value partially sources from their health knowledge exchange behaviours in OHCs. Health knowledge sharing is beneficial to meet patients’ health needs and improve their perception of value. OHC administrators could make policies to encourage more users to engage in OHCs. For example, OHC administrators can optimize OHC design or categorize health knowledge into different domains to make OHCs easy to use.

Second, OHC administrators should be cautious about the use of structural social capital. As our empirical results show, the moderating effects of structural social capital are complex. It weakens the effect of health knowledge seeking but enhances the effect of health knowledge contributing. OHC administrators could lead and encourage users to participate in frequent, diverse and intensive meaningful interactions (eg health knowledge discussion) with knowledgeable health professionals or users.

### 6.3 Limitations

There are several limitations that may affect the findings in this study. First, our sample size is relatively small. Empirical findings might be more robust with a larger sample. Second, we built a concise model that includes three antecedents (ie health knowledge seeking, health knowledge contributing and structural social capital). We did not include the factors such as types of health knowledge,^32^ characteristics of health care,^26^ type of patients’ illnesses and characteristics of OHCs that might influence patients’ perceived value of OHCs. Including these variables, especially the characteristics of health care and OHCs, could capture the impacts of contextual factors and therefore might have interesting findings. We address the lack of examining the impacts of these factors as a limitation of this study. We appeal to scholars to pay more attention to these factors and explore their impacts on patients’ perceived value of OHCs in future studies.

## 7 CONCLUSIONS

We posit that patients’ perceived value of OHCs is influenced by both health knowledge sharing behaviours and environmental factors. We build a model composed of five hypotheses according to SCT and verified it with a sample of 352 valid responses. We have verified that health knowledge seeking and health knowledge contributing behaviours positively influence patients’ perceived value of OHCs; in addition, the impact of health knowledge seeking is greater than the impact of health knowledge contributing. Structural social capital works as a moderator that changes the impacts of patients’ health knowledge seeking and contributing behaviours on their perceived value of OHCs. It provides knowledge seekers more personal channels to seek knowledge directly from OHCs and weakens patients’ perception of value derived from knowledge seeking behaviours in OHCs; meanwhile, it provides knowledge contributors more opportunities to demonstrate their knowledge in OHCs and enhances their perception of value derived from knowledge contributing in OHCs. These findings contribute to the literature on users’ perceived value of OHCs by advancing the understanding of how behavioural factors and environmental factors influence patients’ perceived value of OHCs.

## CONFLICT OF INTEREST

The author declares that there is no conflict of interest.

## Data Availability

The data that support the findings of this study are available on request from the corresponding author. The data are not publicly available due to privacy or ethical restrictions.
